# Individualization as Driving Force of Clustering Phenomena in Humans

**DOI:** 10.1371/journal.pcbi.1000959

**Published:** 2010-10-21

**Authors:** Michael Mäs, Andreas Flache, Dirk Helbing

**Affiliations:** 1 Department of Sociology, Interuniversity Center for Social Science Theory and Methodology, University of Groningen, Groningen, The Netherlands; 2 Chair of Sociology, in particular of Modeling and Simulation, ETH Zurich, Zurich, Switzerland; 3 Santa Fe Institute, Santa Fe, New Mexico, United States of America; 4 Collegium Budapest-Institute for Advanced Study, Budapest, Hungary; University of Washington, United States of America

## Abstract

One of the most intriguing dynamics in biological systems is the emergence of clustering, in the sense that individuals self-organize into separate agglomerations in physical or behavioral space. Several theories have been developed to explain clustering in, for instance, multi-cellular organisms, ant colonies, bee hives, flocks of birds, schools of fish, and animal herds. A persistent puzzle, however, is the clustering of opinions in human populations, particularly when opinions vary continuously, such as the degree to which citizens are in favor of or against a vaccination program. Existing continuous opinion formation models predict “monoculture” in the long run, unless subsets of the population are perfectly separated from each other. Yet, social diversity is a robust empirical phenomenon, although perfect separation is hardly possible in an increasingly connected world. Considering randomness has not overcome the theoretical shortcomings so far. Small perturbations of individual opinions trigger social influence cascades that inevitably lead to monoculture, while larger noise disrupts opinion clusters and results in rampant individualism without any social structure. Our solution to the puzzle builds on recent empirical research, combining the integrative tendencies of social influence with the disintegrative effects of individualization. A key element of the new computational model is an adaptive kind of noise. We conduct computer simulation experiments demonstrating that with this kind of noise a third phase besides individualism and monoculture becomes possible, characterized by the formation of metastable clusters with diversity *between* and consensus *within* clusters. When clusters are small, individualization tendencies are too weak to prohibit a fusion of clusters. When clusters grow too large, however, individualization increases in strength, which promotes their splitting. In summary, the new model can explain cultural clustering in human societies. Strikingly, model predictions are not only robust to “noise”—randomness is actually the central mechanism that sustains pluralism and clustering.

## Author Summary

Modern societies are characterized by a large degree of pluralism in social, political and cultural opinions. In addition, there is evidence that humans tend to form distinct subgroups (clusters), characterized by opinion consensus within the clusters and differences between them. So far, however, formal theories of social influence have difficulty explaining this coexistence of global diversity and opinion clustering. In this study, we identify a missing ingredient that helps to fill this gap: the striving for uniqueness. Besides being influenced by their social environment, individuals also show a desire to hold a unique opinion. Thus, when too many other members of the population hold a similar opinion, individuals tend to adopt an opinion that distinguishes them from others. This notion is rooted in classical sociological theory and is supported by recent empirical research. We develop a computational model of opinion dynamics in human populations and demonstrate that the new model can explain opinion clustering. We conduct simulation experiments to study the conditions of clustering. Based on our results, we discuss preconditions for the persistence of pluralistic societies in a globalizing world.

## Introduction

Many biological systems exhibit collective patterns, which emerge through simple interactions of large numbers of individuals. A typical example is agglomeration phenomena. Such clustering dynamics have been found in systems as different as bacterial colonies [1], gregarious animals like cockroaches [2], fish schools [3], flocks of birds [4], and animal groups [5]. Similar phenomena are observed in ecosystems [6] and human populations, as examples ranging from the formation of pedestrian groups [7] to the formation of urban agglomerations demonstrate [8], [9].

Recently, numerous studies on the structure of human interaction networks [10]–[12] demonstrated that clustering is *not* restricted to physical or geographical space. For instance, clustering has been extensively studied in networks of email communication [13], phone calls [12], scientific collaboration [14] and sexual contacts [15]. It is much less understood, however, how and what conditions clustering patterns emerge in behavioral or opinion space. Empirical studies suggest that opinions differ globally [16], [17], while they cluster locally within geographical regions [18], socio-demographic groups [19], or Internet communities [20]. In addition, research on dynamics in work teams demonstrates that even groups of very small size often show high opinion diversity and can even suffer from opinion polarization [21], [22].

Opinion clustering is defined as the co-existence of distinct subgroups (clusters) of individuals with similar opinions, while opinions in different subgroups are relatively large. The gaps in our theoretical understanding of opinion clustering are pressing since both local consensus and global diversity are precarious. On the one hand, cultural diversity may get lost in a world where people are increasingly exposed to influences from mass media, Internet communication, interregional migration, and mass tourism, which may promote a universal monoculture [23], [24], as the extinction of languages suggests [25]. On the other hand, increasing individualization threatens to disintegrate the social structures in which individuals are embedded, with the possible consequence of the loss of societal consensus [26], [27]. This is illustrated by the recent debate on the decline of social capital binding individuals into local communities [28].

Early formal models of social influence imply that monoculture is unavoidable, unless a subset of the population is perfectly cut off from outside influences [29]. Social isolation, however, appears questionable as explanation of pluralism. In modern societies, distances in social networks are quite short on the whole, and only relatively few random links are required to dramatically reduce network distance [10].

**Figure 1:**
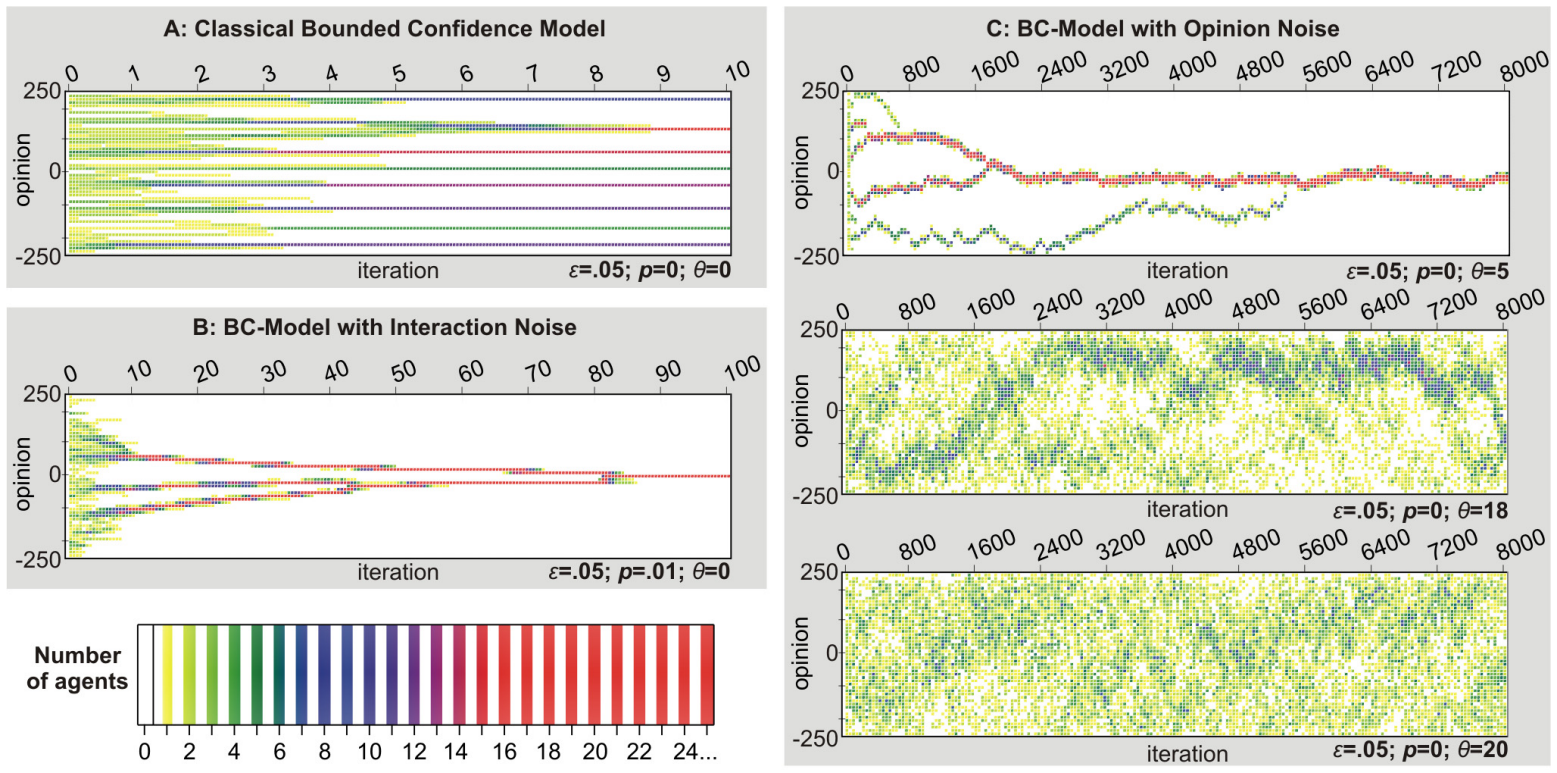
Opinion dynamics produced by the bounded confidence (BC) model [33] with and without noise. Populations consist of 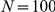 agents. Opinions vary between −250 and 250. Initial opinions are uniformly distributed. For visualization, the opinion scale is divided into 50 bins of equal size. Color coding indicates the relative frequency of agents in each bin. (A) Dynamics of the *BC-model without noise* [33] over 10 iterations (Each iteration consists of 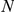 simulation events 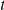.). At each simulation event, one agent's opinion is replaced by the average opinion of those other agents who hold opinions 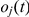 within the focal agent's confidence interval (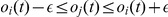). For 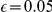, one finds several homogeneous clusters, which stabilize when the distance between all clusters exceeds the confidence threshold 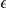. (B) Computer simulation of the same BC-model, but considering *interaction noise*. Agents that would otherwise not have been influential, now influence the focal agent's opinion with a probability of 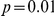. This small noise is sufficient to eventually generate monoculture. (C) Simulation of the BC-model with opinion noise. After each opinion update, a random value drawn from a normal distribution with an average of zero and a standard deviation of 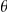 (abbreviated by 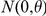) is added to the opinion. For weak opinion noise (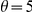), one cluster is formed, which carries out a random walk on the opinion scale. When the opinion noise is significantly increased (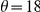), there is still one big cluster, but many separated agents exist as well (cf. Fig. 4). With even stronger opinion noise (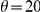), the opinion distribution becomes completely random.

Aiming to explain pluralism, researchers have incorporated the empirically well-supported observation of “homophily”, i.e. the tendency of “birds of a feather to flock together” [30], [31], into formal models of social influence [32]. These models typically assume “bounded confidence” (BC) in the sense that only those individuals interact, whose opinions do not differ more than a given threshold level [33], [34]. As Fig. 1A illustrates, BC generates opinion clustering, a result that generalizes to model variants with categorical rather than continuous opinions [32], [35]. However, clustering in the BC-model is sensitive to “interaction noise”: A small random chance that agents may interact even when their opinions are not similar, causes monoculture again (see Fig. 1B).

To avoid this convergence of opinions, it was suggested that individuals would separate themselves from negatively evaluated others [19], [36], [37]. However, recent empirical results do not support such “negative influence” [38]. Scientists also tried to avoid convergence by “opinion noise”, i.e. random influences, which lead to arbitrary opinion changes with a small probability. Assuming uniformly distributed opinion noise [39] leads to sudden, large, and unmotivated opinion changes of individuals, while theories of social integration [26], [27], [40], [41] and empirical studies of individualization [42], [43] show a tendency of incremental opinion changes rather than arbitrary opinion jumps. Incremental opinion changes, however, tend to promote monoculture, even in models with categorical rather than continuous opinions [44]. Fig. 1 demonstrates that adding a “white noise” term (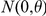) to an agent's current opinion in the BC model fails to explain opinion clustering. Weak opinion noise (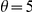) triggers convergence cascades that inevitably end in monoculture. Stronger noise restores opinion diversity, but not clustering. Instead, diversity is based on frequent individual deviations from a predominant opinion cluster (for 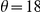). However, additional clusters cannot form and persist, because opinion noise needs to be strong to separate enough agents from the majority cluster—so strong that randomly emerging smaller clusters cannot stabilize.

In conclusion, the formation of persistent opinion clusters is such a difficult puzzle that all attempts to explain them had to make assumptions that are difficult to justify by empirical evidence. The solution proposed in the following, in contrast, aims to reconcile model assumptions with sociological and psychological research. The key innovation is to integrate another decisive feature into the model, namely the “striving for uniqueness” [42], [43]. While individuals are influenced by their social environment, they also show a desire to increase their uniqueness when too many other members of society hold similar opinions. We incorporate this assumption as a white noise term in the model. However, in contrast to existing models we assume that noise strength is not constant but *adaptive*. To be precise, we assume that the impact of noise on the opinion of an individual is the stronger the less unique the individual's opinion is compared to the other members of the population. Consumer behavior regarding fashions illustrates the adaptability of opinion noise: When new clothing styles are adopted by some people, they often tend to be imitated by others with similar spirit and taste (the “peer group”). However, when imitation turns the new style into a norm, people will seek to increase their uniqueness. This will sooner or later lead some individuals to invent new ways to dress differently from the new norm.

Adaptive noise creates a dynamic interplay of the integrating and disintegrating forces highlighted by Durkheim's classic theory of social integration [26]. Durkheim argued that integrating forces bind individuals to society, motivating them to conform and adopt values and norms that are similar to those of others. But he also saw societal integration as being threatened by disintegrating forces that foster individualization and drive actors to differentiate from one another [27], [40], [41]. The “Durkheimian opinion dynamics model” proposed in the following can explain pluralistic clustering for the case of continuously varying opinions, although it incorporates all the features that have previously been found to *undermine* clustering: (1) a fully connected influence network, (2) absence of bounded confidence, (3) no negative influence, and (4) white opinion noise. From a methodological viewpoint, our model builds on concepts from statistical physics, namely the phenomenon of “nucleation” [45], illustrated by the formation of water droplets in supersaturated vapor. However, by assuming adaptive noise, we move beyond conventional nucleation models. The model also resembles elements of Interacting Particle Systems [46] like the voter model and the anti-voter model [47]–[50] which have been used to study dynamics of discrete opinions (“pro” and “contra”). However, we focus here on *continuous* opinions like the degree to which individuals are in favor of or against a political party.

Computational simulation experiments reveal that, despite the continuity of opinions in our model, it generates pluralism as an intermediate phase between monoculture and individualism. When the integrating forces are too strong, the model dynamics inevitably implies monoculture, even when the individual opinions are initially distributed at random. When the disintegrating forces prevail, the result is what Durkheim called “anomie”, a state of extreme individualism without a social structure, even if there is perfect consensus in the beginning. Interestingly, there is no sharp transition between these two phases, when the relative strength of both forces is changed. Instead, we observe an additional, intermediate regime, where opinion clustering occurs, which is independent of the initial condition. In this regime, adaptive noise entails robust pluralism that is stabilized by the adaptability of cluster size. When clusters are small, individualization tendencies are too weak to prohibit a fusion of clusters. However, when clusters grow large, individualization increases in strength, which triggers a splitting into smaller clusters (“fission”). In this way, our model solves the cluster formation problem of earlier models. While in BC models, white noise causes either monoculture or fragmentation (Fig. 1C), in the Durkheimian opinion dynamics model proposed here, it *enables* clustering. Therefore, rather than *endangering* cluster formation, noise supports it. In the following, we describe the model and identify conditions under which pluralism can flourish.

## Model

The model has been elaborated as an agent-based model [51] addressing the opinion dynamics of interacting individuals. The simulated population consists of 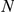 agents 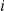, representing individuals, each characterized by an opinion 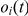 at time 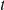. The numerical value for the opinion varies between a given minimum and maximum value on a metric scale. We use the term “opinion” here, for consistency with the literature on social influence models. However, 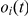 may also reflect behaviors, beliefs, norms, customs or any other cardinal cultural attribute that individuals consider relevant and that is changed by social influence. The dynamics is modeled as a sequence of events. Every event 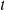 the computer randomly picks an agent 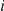 and changes the opinion 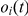 by the amount
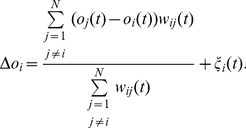
(1)The first term on the rhs of Eq. [1] models the integrating forces of Durkheim's theory. Technically, agents tend to adopt the weighted average of the opinions 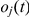 of all other members 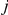 of the population. Implementing homophily, the social influence 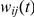 that agent 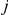 has on agent 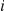 is the stronger, the smaller their opinion distance 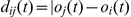 is. Formally, we assume
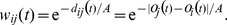
(2)The parameter 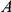 represents the range of social influence of agents. For small positive values of 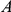, agents are very confident in their current opinion and are mainly influenced by individuals who hold very similar opinions, while markedly distinct opinions have little impact. The higher 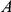 is, however, the more are agents influenced by individuals with considerably different opinions and the stronger are the integrating forces in our Durkheimian theory.

The *dis*integrating forces on the opinion of agent 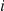 are modeled by a noise term 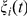. Specifically, the computer adds a normally distributed random value 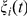 (“white noise”) to the first term on the rhs of Eq. [1]. While we assume that the mean value of the random variable 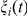 is zero, the standard deviation has been specified as
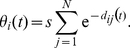
(3)


The larger the standard deviation, the stronger are the individualization tendencies of an agent. Following Durkheim's theory, equation [3] implements noise in an *adaptive* way: Accordingly, an agent's striving for individualization is weak, if there are only a few others with similar opinions. Under such conditions, there is no need to increase distinctiveness. However, if many others hold a similar opinion, then individuals are more motivated to differ from others.

By including the focal agent 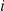 in the sum of Eq. [3], we assume that there is always some degree of opinion noise, even when agent 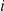 holds a perfectly unique opinion. These fluctuations may have a variety of reasons, such as misjudgments, trial-and-error behavior, or the influence of exogenous factors on the individual opinion. Furthermore, this assumption reflects Durkheim's notion that the seeking for uniqueness is a fundamental feature of human personality, which cannot be suppressed completely [26], [52].

We use the parameter 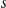 of Eq. [3] to vary the strength of the disintegrating forces in society. The higher the value of 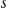, the higher is the standard deviation of the distribution, from which 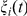 is drawn, and the stronger are the disintegrating forces. Finally, to keep the opinions of the agents within the bounds of the opinion scale, we set the value of 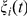 to zero, if the bounds of the opinion space would be left otherwise.

## Results

We have studied the Durkheimian opinion dynamics model with extensive computer simulations, focusing on relatively small populations (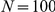), because in this case it is reasonable to assume that all members may interact with each other. For bigger populations one would have to take into account the topology of the social interaction network as well. Such networks would most likely consist of segregated components (“communities”), which are not or only loosely connected with each other [12]–[15]. Existing social influence models can explain how under such conditions each community develops its own shared opinion (see Fig. 1A). However, according to these models opinion clustering is only stable when there is no interaction between communities [29], [33], an assumption that appears *not* to be empirically correct in an increasingly connected world. Therefore, we focus on a setting for which the lack of connectedness is guaranteed to be excluded as explanation of clustering and study model dynamics in relatively small and complete interaction networks.

**Figure 2:**
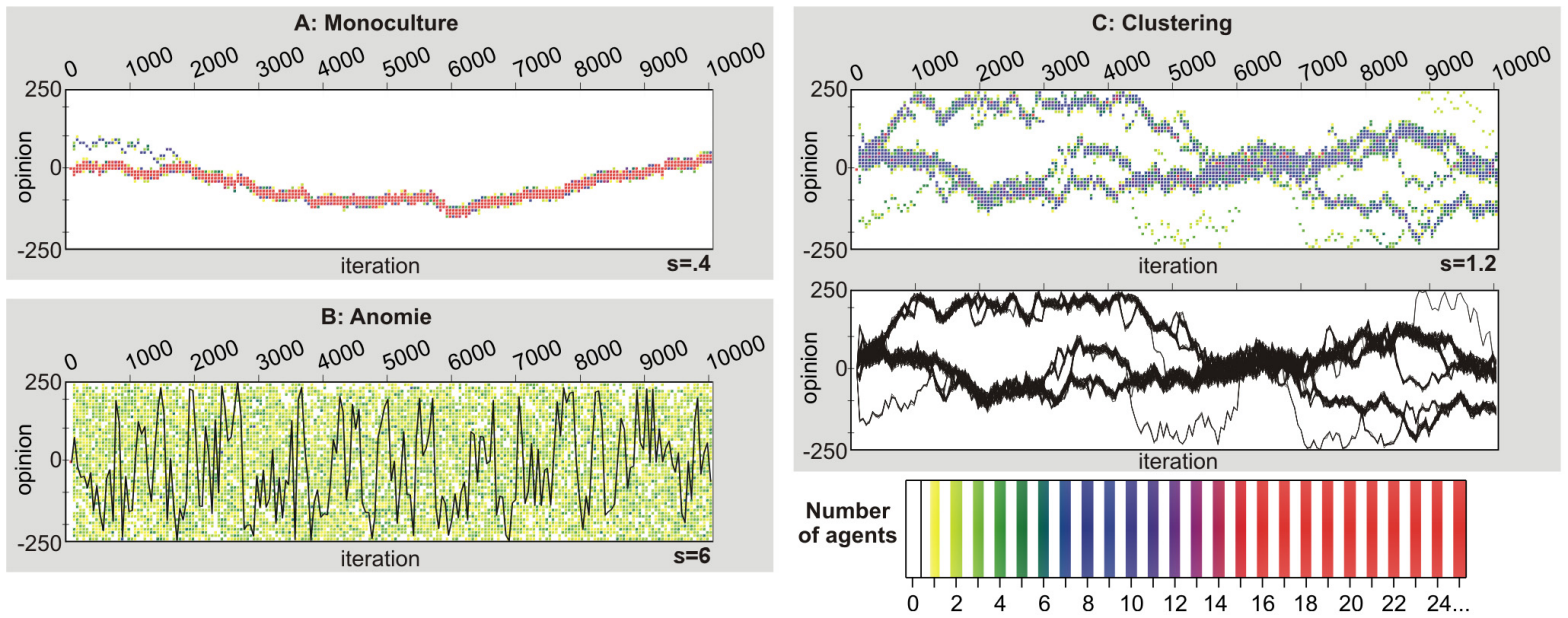
Opinion trajectories of three representative simulation runs with 100 agents generated by the Durkheimian model. In all three runs, the opinions are restricted to values between −250 and 250, and all agents hold the same opinion initially (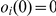 for all 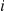). In all runs, we assume the same social influence range 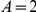, but vary the strength 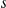 of the disintegrating force. (A) Monoculture, resulting in the case of a weak disintegrating force (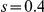). Agents do not hold perfectly identical opinions, but the variance is low. We studied dynamics over 10.000 iterations (Each iteration consists of 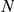 simulation events 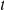.). (B) Anomie (i.e. extreme individualism), generated by a very strong disintegrating force (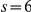). Agents spread over the complete opinion scale. The black line represents the time-dependent opinion of a single, randomly picked agent, showing significant opinion changes over time, which is in contrast to the collective opinion formation dynamics found in the monocultural and pluralistic cases (A) and (B). (C) For a moderate disintegrating force (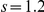), the population quickly disintegrates into clusters. As long as these clusters are small, they are metastable. However, clusters perform random walks and can merge (e.g. around iteration 5500). As the disintegrating force grows with the size of a cluster, big clusters eventually split up into subclusters (e.g. around iteration 7000). The additional graph, in which each agent's opinion trajectory is represented by a solid black line, is an alternative visualization of the simulation run with 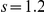. It shows that the composition of clusters persists over long time periods.

To illustrate the model dynamics, Fig. 2 shows three typical simulation runs for different strengths 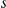 of disintegrating forces, while the strength 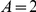 of the integrating force is kept constant. In each run, all agents start with an opinion in the middle of the opinion scale (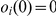), i.e. conformity. This is an initial condition for which the classical BC-model does not produce diversity. Fig. 2A shows typical opinion trajectories for a population in which the integrating forces are much stronger than the disintegrating forces. Consequently, the population develops collective consensus, i.e. the variation of opinions remains small, even though not all agents hold exactly the same opinion. Triggered by the random influences 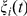, the average opinion performs a characteristic random walk.

When the disintegrating force prevails, the pattern is strikingly different. Fig. 2B shows that for large noise strengths 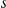, the initial consensus breaks up quickly, and the agents' opinions are soon scattered across the entire opinion space.

Simulation scenarios A and B are characteristic for what Durkheim referred to as states of social cohesion and of anomie. Interestingly, however, pluralism arises as a third state in which several opinion clusters form and coexist. Fig. 2C shows a typical simulation run, where the adaptive noise maintains pluralism despite the antagonistic impacts of integrating and disintegrating forces—in fact *because* of this. In the related region of the parameter space, disintegrating forces prevent global consensus, but the integrating forces are strong enough to also prevent the population from extreme individualization. This is in pronounced contrast to what we found for the BC-model with strong noise (Fig. 1C). Instead, we obtain a number of coexisting, metastable clusters of a characteristic, parameter-dependent size. Each cluster consists of a relatively small number of agents, which keeps the disintegrating forces in the cluster weak and allows clusters to persist. (Remember that the tendency of individualization according to Eq. [3] increases, when many individuals hold similar opinions.) However, due to opinion drift, distinct clusters may eventually merge. When this happens, the emergent cluster becomes unstable and will eventually split up into smaller clusters, because disintegrating forces increase in strength as a cluster grows.

**Figure 3:**
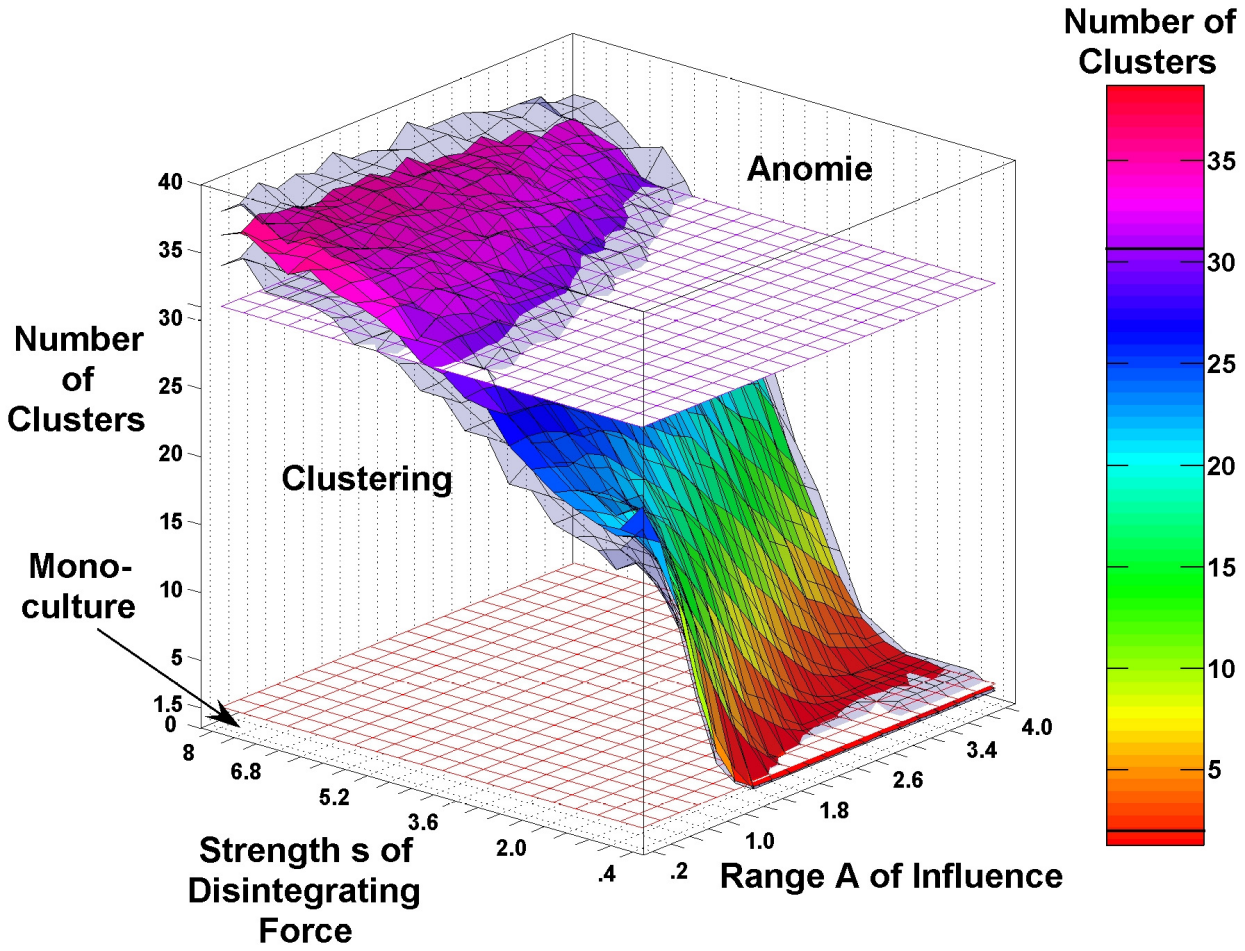
Conditions of clustering, monoculture and Anomie. The figure shows the dependence of the average number of clusters in the Durkheimian model on the strength 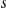 of the disintegrating force and the range 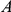 of social influence. To generate it, we conducted computer simulations with 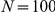 agents, starting with initial consensus (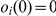 for all 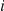). We restricted opinions to values between −250 and 250. We varied the strength 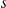 of the disintegrating force between 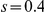 and 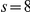 in steps of 0.4. 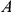 varied between 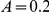 and 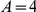 in steps of 0.2. For each parameter combination, we conducted 100 independent replications and assessed the average number of clusters formed after 250,000 iterations (see 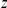-axis and the color scale). The two transparent (gray) surfaces depict the inter-quartile range, which indicates a small variance in the number of clusters (and also typical cluster sizes) for each parameter combination. The horizontal grids indicate the borders of the three phases, as defined by us. An average cluster size below 1.5 indicates monoculture. Values between 1.5 and 31 reflect clustering. Finally, values above 31 correspond to opinion distributions that cannot be distinguished from random ones and represent a state of anomie.

Strikingly, the state of diversity, in which several opinion clusters can coexist, is not restricted to a narrow set of conditions under which integrating and disintegrating forces are balanced exactly. Fig. 3 demonstrates that opinion clusters exist in a significant area of the parameter space, i.e. the clustering state establishes another phase, which is to be distinguished from monoculture and from anomie.

To generate Fig. 3, we conducted a simulation experiment in which we varied the influence range 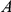 and the strength 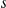 of the disintegrating force. For each parameter combination, we ran 100 replications and measured the average number of clusters that were present after 250,000 iterations. To count the number of clusters in a population, we ordered the 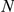 agents according to their opinion. A cluster was defined as a set of agents in adjacent positions such that each set member was separated from the adjacent set members by a maximum of 5 scale points ( = opinion range/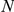). Fig. 3 shows that, for large social influence ranges 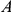 and small noise strengths 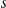, the average number of clusters is below 1.5, reflecting monoculture in the population. In the other extreme, i.e. for a small influence range 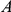 and large noise strengths 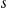, the resulting distribution contains more than 31 clusters, a number of clusters that cannot be distinguished from purely random distributions. Following Durkheim, we have classified such cases as anomie, i.e. as the state of extreme individualism. Between these two phases, there are numerous parameter combinations, for which the number of clusters is higher than 1.5 and clearly smaller than in the anomie phase. This constitutes the clustering phase. Fig. 3 also shows that, for each parameter combination, there is a small variance in the number of clusters, which is due to a statistical equilibrium of occasional fusion and fission processes of opinion clusters (see Fig. 2C).

**Figure 4:**
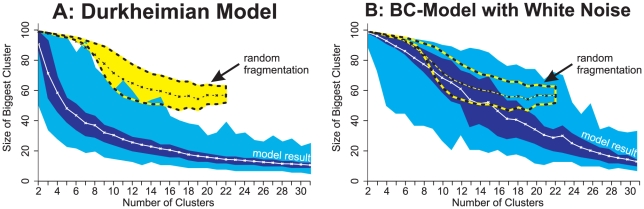
Comparison of the (A) Durkheimian model and (B) the noisy BC-model. Figures plot the size of the biggest cluster versus the number of clusters and compare it to the case of random fragmentation in all simulation runs that resulted in more than one and less than 32 clusters. Fig. 4A is based on the simulation experiment with the Durkheimian model underlying Fig. 3. Fig. 4B is based on an experiment with the BC-model [33] where we varied the bounded-confidence level 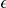 between 0.01 and 0.15 in steps of 0.02 and the noise level 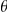 between 5 and 50 in steps of 5. We conducted 100 replications per parameter combination and measured the number of clusters and the size of the biggest cluster after 250,000 iterations. White solid lines represent the average size of the biggest cluster. The dark blue area shows the respective interquartile range and the light blue area the complete value range. For comparison, we generated *randomly fragmented opinion distributions* of 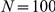 agents where 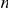 agents hold random opinions (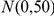) and the remaining 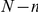 agents hold opinion 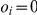 and form one big cluster. We varied the value of 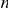 between 0 and 100 in steps of 1 and generated 1000 distributions per condition. The average size of the biggest cluster of the resulting distributions is shown by the thin yellow-black line. (The curve stops at 22, since this is the highest number of clusters generated.) The bold yellow-black lines represent the related interquartile range. We find that the value range of the Durkheimian model (blue area) hardly overlaps with the interquartile range of the fragmented distributions (yellow area). This demonstrates that the Durkheimian model shows clustering rather than fragmentation. In contrast, Fig. 4B illustrates that the distributions of the noisy BC-model and the results for random fragmentation overlap.

The same results were found, when starting the computer simulations with a uniform opinion distribution. This demonstrates that the simulations were run long enough (250,000 iterations) to obtain reliable results. It also suggests that clustering is an attractor in the sense that the model generates clustering independent of the initial distribution of opinions. In addition, we performed additional statistical tests with the simulation outcomes to make sure that the existence of clusters in our model indeed indicates pluralism and not fragmentation, a state in which a population consists of one big cluster and a number of isolated agents (see Fig. 4). To illustrate, Fig. 4A plots the size of the biggest cluster in the population versus the number of clusters (see the blue areas). For comparison, the yellow area depicts the corresponding distribution for randomly fragmented opinion distributions. The figure shows that the distributions hardly overlap and that the Durkheimian model generates clustering rather than fragmentation. In clear contrast, Fig. 4B reveals that the opinion distributions generated by the noisy BC-model are fragmented and *not* clustered. Finally, to exclude that results have been influenced by floating point inaccuracies [53] we conducted simulation experiments with the restriction that influence weights 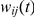 could not adopt values smaller than 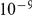. All results could be replicated.

## Discussion

The phenomenon of self-organized clustering phenomena in biological and social systems is widespread and important. With the advent of mathematical and computer models for such phenomena, there has been an increasing interest to study them also in human populations. The work presented here focuses on resolving the long-standing puzzle of opinion clustering.

The emergence and persistence of pluralism is a striking phenomenon in a world in which social networks are highly connected and social influence is an ever present force that reduces differences between those who interact. We have developed a formal theory of social influence that, besides anomie and monoculture, shows a third, pluralistic phase characterized by opinion clustering. It occurs, when all individuals interact with each other and noise prevents the convergence to a single opinion, despite homophily.

Our model does not assume negative influence, and it behaves markedly different from bounded confidence models, in which white opinion noise produces fragmentation rather than clustering. Furthermore, our model does not rely on the problematic assumption of classical influence models that agents are forevermore cut-off from influence by members of distinct clusters. In order to demonstrate this, we studied model predictions in a setting where all members of the population interact with each other. However, empirical research shows that opinion clustering tends to coincide with clustered network structures [20] and spatial separation [18]. It would therefore be natural to generalize the model in a way that it also considers the structure of real social networks. Such a model is obtained by replacing the values 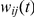 by 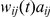, where 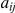 are the entries of the adjacency matrix (i.e. 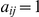, if individuals 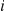 and 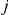 interact, otherwise 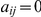). Then, the resulting opinion clusters are expected to have a broad range of different sizes, similar to what is observed for the sizes of social groups.

Our model highlights the functional role that “noise” (randomness, fluctuations, or other sources of variability) plays for the organization of social systems. It furthermore shows that the combination of two mechanisms (deterministic integrating forces and stochastic disintegrating forces) can give rise to new phenomena. We also believe that our results are meaningful for the analysis of the social integration of our societies. According to Durkheim's theory of the development of societies [26], traditional human societies are characterized by “mechanical solidarity”. In these societies, individuals are strongly integrated in very homogeneous communities which exert strong influence on the behavior and opinions of individuals. According to Durkheim, however, these regulating social structures dissolve as societies turn modern. In addition, Durkheim [26] and contemporary social thinkers [27] argue that in modern and globalized societies individuals are increasingly exposed to disintegrating forces, which foster individualization [26]. As a consequence, the social forces which let individuals follow societal norms may lose their power to limit individual variation. Durkheim feared that the high diversity could disintegrate societies as they modernize [26]. That is, extreme individualization in modern societies may obstruct the social structures that traditionally provided social support and guidance to individuals.

Today, modern societies are highly diverse, but at the same time they are far from a state of disintegration as foreseen by Durkheim. He argued that this is possible if societies develop what he called “organic solidarity”. In this state societies are highly diverse but at the same time the division of labor creates a dense web of dependencies which integrate individuals into society and generate sufficient moral and social binding [26]. Strikingly, our formal model of Durkheim's theory revealed another possibility which does not require additional integrating structures like the division of labor: Besides monoculture and anomie, there is a third, pluralistic clustering phase, in which individualization prevents overall consensus, but at the same time, social influence can still prevent extreme individualism. The interplay between integrating and disintegrating forces leads to a plurality of opinions, while metastable subgroups occur, within which individuals find a local consensus. Individuals may identify with such subgroups and develop long-lasting social relationships with similar others. Therefore, they are not isolated and not without support or guidance, in contrast to the state of disintegration that Durkheim was worried about.

We have seen, however, that pluralism and cultural diversity require an approximate balance between integrating and disintegrating forces. If this balance is disturbed, societies may drift towards anomie or monoculture. It is, therefore, interesting to ask how the current tendency of globalization will influence society and cultural dynamics. The Internet, interregional migration, and global tourism, for example, make it easy to get in contact with members of distant and different cultures. Previous models [24], [35] suggest that this could affect cultural diversity in favor of a monoculture. However, if the individual striving for uniqueness is sufficiently strong, formation of diverse groups (a large variety of international social communities) should be able to persist even in a globalizing world. In view of the alternative futures, characterized by monoculture or pluralism, further theoretical, empirical, and experimental research should be performed to expand our knowledge of the mechanisms that will determine the future of pluralistic societies.
